# Durable Superhydrophobic Composite Coating Based on Hydrangea-like SiO_2_ Nanoparticles with Excellent Performance in Anticorrosion, Drag Reduction, and Antifouling

**DOI:** 10.3390/ma18153443

**Published:** 2025-07-23

**Authors:** Yuhao Xue, Yamei Zhao, Xiaoqi Gu, Mengdan Huo, Kunde Yang, Mingyu Liu, Sixian Fan, Maoyong Zhi

**Affiliations:** 1 Department of Chemical Engineering, School of Environmental and Chemical Engineering, Xi’an Polytechnic University, Xi’an 710048, China; 18192575313@163.com (Y.X.); 18182608382@163.com (X.G.); 13369182020@163.com (M.H.); syrxhnhjl@163.com (M.L.); 17659717195@163.com (S.F.); 2 Sichuan Key Technology Engineering Research Center for All-Electric Navigable Aircraft, Civil Aviation Flight University of China, Guanghan 618307, China; zhimaoyong@cafuc.edu.cn; 3 Ocean Institute, Northwestern Polytechnical University, Taicang 215400, China; ykdzym@nwpu.edu.cn

**Keywords:** superhydrophobic coating, durability, durability anti-corrosion, reduce drag, antifouling

## Abstract

Superhydrophobic coatings possess distinct wettability characteristics and hold significant potential in metal corrosion protection and underwater drag reduction. However, their practical application is often hindered by poor durability arising from the fragility of their micro/nanostructured surface roughness. In this study, a durable superhydrophobic coating featuring a hierarchical, hydrangea-like micro/nanostructure was successfully fabricated on an aluminum alloy substrate via a simple one-step cold-spraying technique. The coating consisted of hydrangea-shaped SiO_2_ nanoparticles modified with 1H,1H,2H,2H-perfluorodecyltrimethoxysilane (PFDT) to produce multiscale roughness, while epoxy resin (EP) served as the binding matrix to enhance mechanical integrity. The hydrangea-like SiO_2_ nanostructures were characterized by solid cores and wrinkled, petal-like outgrowths. This unique morphology not only increased the surface roughness but also provided more active sites for air entrapment, thereby enhancing the coating’s overall performance. The h-SiO_2_@PFDT-EP composite coating exhibited excellent superhydrophobicity, with a WCA of 170.1° ± 0.8° and a SA of 2.7° ± 0.5°. Durability was evaluated through sandpaper abrasion, tape peeling, acid and alkali immersion, artificial weathering, and salt spray tests. The results demonstrated that the coating retained stable superhydrophobic performance under various environmental stresses. Compared with bare 6061 aluminum and EP coatings, its corrosion current density was reduced by four and three orders of magnitude, respectively. Furthermore, the coating achieved a maximum drag-reduction rate of 31.01% within a velocity range of 1.31–7.86 m/s. The coating also displayed excellent self-cleaning properties. Owing to its outstanding durability, corrosion resistance, and drag-reducing capability, this one-step fabricated superhydrophobic coating showed great promise for applications in marine engineering and defense.

## 1. Introduction

The drag reduction performance during high-speed motion [1,2,3] and the corrosion resistance of metals [4,5,6,7] were regarded as two critical factors for marine equipment. Superhydrophobic surfaces had attracted significant attention from both academia and industry owing to their unique wettability characteristics [8,9]. A superhydrophobic surface was typically characterized by a water contact angle (WCA) exceeding 150° and a sliding angle (SA) below 10°. Superhydrophobic coatings had been applied in self-cleaning [10,11], corrosion resistance [12,13], drag reduction [14], anti-icing [15,16], and oil–water separation [17]. The construction of superhydrophobic surfaces has mainly relied on suitable surface microstructures and low surface energy. Through the formation of hierarchical microstructures and the reduction of surface energy, a stable air layer could be formed at the liquid–solid interface, which reduced the contact area between water and the substrate, thereby enhancing corrosion resistance, fouling resistance, hydrodynamic drag reduction, and the operational lifespan of marine devices [18,19,20].

In recent years, research has predominantly focused on the fabrication of superhydrophobic coatings with engineered surface structures on metal substrates such as aluminum alloys, magnesium alloys, and stainless steel, aiming to improve corrosion resistance and reduce hydrodynamic drag [20,21]. Microscale surface roughness and ultra-low surface energy were considered essential for achieving high-performance superhydrophobicity [22,23,24]. At present, two primary strategies have been widely employed to fabricate such coatings. The first strategy involves the in situ construction of rough microstructures directly on low-surface-energy materials through techniques such as laser etching, anodization, and hydrothermal synthesis [25,26,27,28,29]. For instance, Wang et al. [26] fabricated continuous “armor-like” microstructures embedded with nanoscale features on silicon wafers that maintained superhydrophobicity even after extensive abrasion. Similarly, Huang et al. [27] produced Mg–Al layered double hydroxides on magnesium alloys via continuous etching, followed by surface modification with octadecyltrimethoxysilane, resulting in contact angles greater than 150° and sliding angles below 10°. However, these techniques typically required sophisticated equipment and stringent experimental conditions, leading to high costs and limited scalability.

In contrast, the second strategy utilized micro/nano-structured superhydrophobic particles to construct multiscale surface roughness through methods such as spraying, spin coating, dip-coating, or immersion [30,31,32,33]. For example, Zhang et al. [30] sequentially sprayed micron-scale polypropylene and nano-scale SiO_2_ suspensions onto acrylic substrates to construct a hierarchical bilayer that retained superhydrophobicity under water jets at 9.5 m/s and a hydrostatic pressure of 0.1 MPa. Wu et al. [31] prepared Al_2_O_3_/epoxy nanocomposites by reverse injection spraying, resulting in wear-resistant coatings that endured over 500 abrasion cycles. Nanoparticles such as SiO_2_ and TiO_2_ have been widely employed to introduce surface roughness in the construction of superhydrophobic coatings [34,35]. With the development of nanotechnology, mesoporous silica nanoparticles have received increasing attention due to their large specific surface area, tunable pore size, and highly ordered porosity, making them highly suitable for functional surface engineering [36,37,38].

This study aimed to improve the hydrophobicity and durability of superhydrophobic surfaces by tailoring the morphology of silica and integrating epoxy resin (EP) through a one-step cold-spraying technique. Hydrangea-shaped SiO_2_ nanoparticles were synthesized to endow the coating surface with pronounced hierarchical roughness and enhanced mechanical strength. These h-SiO_2_ nanoparticles were modified with PFDT to obtain h-SiO_2_@PFDT particles, which were then combined with EP and deposited onto 6061 aluminum alloy to form the h-SiO_2_@PFDT-EP superhydrophobic coating. The surface morphology, chemical composition, wettability, and roughness of the coating were systematically characterized. Durability was evaluated through a series of assessments, including sandpaper abrasion, immersion in acidic and alkaline solutions, artificial weathering, and salt spray exposure. Additionally, electrochemical testing and drag-reduction experiments were conducted to investigate the corrosion resistance and flow resistance properties. The scalable and single-step fabrication method demonstrated strong potential for practical applications in marine transportation, underwater surveying, and related fields.

## 2. Experiment

### 2.1. Materials

Silicic acid, hexadecyl trimethyl ammonium bromide (CTAB), triethanolamine (TEA), sodium salicylate (NaSal), EP, an aliphatic amine curing agent (amine value: 600–700 mg KOH/g; viscosity: 80–150 mPa·s), and PFDT were purchased from Shanghai Macklin Biochemical Co., Ltd., Shanghai, China. Absolute ethanol, ethyl acetate, sodium hydroxide, concentrated hydrochloric acid, sodium chloride, and graphite powder were obtained from Tianjin Fuyu Fine Chemical Co., Ltd., Tianjin, China. All reagents were of analytical grade and were used without further purification. The 6061 aluminum alloy (composition provided in Table 1) was sourced from Xinghua Puer Metal Materials Co., Ltd., Xinghua, China. The deionized water used throughout the experiments was produced using an ultrapure water purification system.

### 2.2. Synthesis of the Hydrangea-like Mesoporous SiO_2_ Nanoparticles

Spherical SiO_2_ nanoparticles with a hydrangea-like mesoporous structure were synthesized based on a previously reported method. In a 250.00 mL round-bottom flask, 0.07 g of TEA, 0.17 g of NaSal, and 25.00 mL of deionized water were added. Varying amounts of CTAB were then introduced and the mixture magnetically stirred at 80 °C and 400 rpm for 20 min. After surface bubbles had dissipated, 4.00 mL of TEOS was added dropwise, leading to increasing turbidity and the gradual formation of a milky white gel. Stirring was continued at 80 °C and 400 rpm for an additional 2 h to ensure complete reaction.

Following synthesis, the product was separated by high-speed centrifugation (8000 rpm, 20 min) and washed twice with alternating deionized water and ethanol to remove residual reactants. The resulting material was subsequently dispersed in a hydrochloric acid–ethanol solution (HCl:ethanol = 1:9) and stirred at 60 °C for 18 h to extract the CTAB template. Finally, the solid product was dried in a vacuum oven at 60 °C for 12 h to yield the hydrangea-like mesoporous SiO_2_ nanoparticles.

### 2.3. Preparation of h-SiO_2_@PFDT Superhydrophobic Nanoparticles

SiO_2_@PFDT nanoparticles were synthesized based on a previously reported method. In a beaker, 2.00 g of hydrangea-like mesoporous SiO_2_ nanoparticle powder was dispersed in 4.00 mL of distilled water and 36.00 mL of anhydrous ethanol. The mixture was sonicated for 30 min, followed by the addition of 0.39 mL of PFDT pre-diluted in 18.00 mL of ethanol and 2.00 mL of water. The resulting suspension was magnetically stirred at 90 °C for 6 h to complete the surface modification. The modified product was collected by centrifugation at 2000 rpm for 5 min and washed three times, alternating between distilled water and anhydrous ethanol. It was then dried in an oven at 60 °C for 6 h and designated as h-SiO_2_@PFDT.

### 2.4. Preparation of h-SiO_2_@PFDT-EP Coating

At room temperature, 0.70 g of h-SiO_2_@PFDT nanoparticles was dispersed in 7.00 mL of ethyl acetate and sonicated for 15 min to form a homogeneous suspension. Separately, 3.00 mL of ethyl acetate was mixed with preheated 25 wt.% EP binder, and an amine curing agent was added dropwise. This mixture was sonicated for 5 min to obtain a uniform and transparent solution. The epoxy solution was then added dropwise into the h-SiO_2_@PFDT suspension under magnetic stirring and allowed to react at room temperature for 2 h, yielding the h-SiO_2_@PFDT-EP superhydrophobic coating.

**Figure 1:**
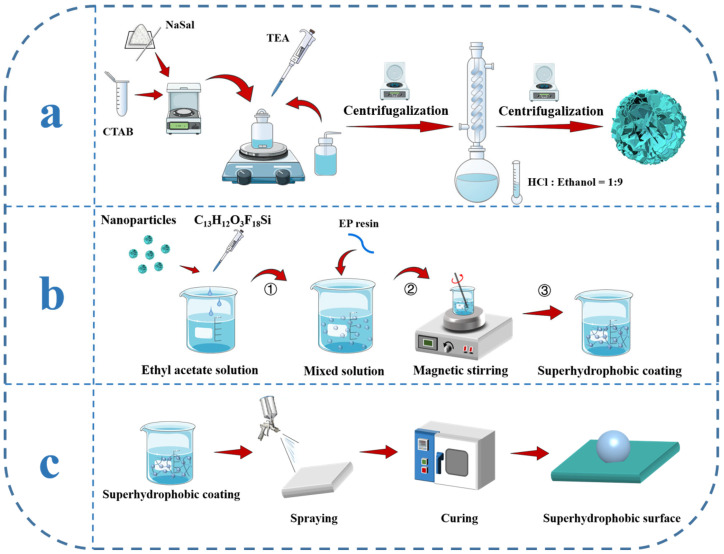
Schematic representation of the preparation process of the h-SiO_2_@PFDT-EP superhydrophobic coating.

The aluminum substrate was cleaned by sequential rinsing with ethanol and deionized water, followed by air-drying. The prepared h-SiO_2_@PFDT-EP coating was applied to the substrate using an HD-407 spray gun (HD-407 spray gun was obtained from Kafwell (Hangzhou) Industrial Co., Ltd., Hangzhou, China) equipped with a 0.30 mm diameter nozzle. The spray velocity was maintained at approximately 5.00 cm·s^−1^, and the spray volume was controlled at 150.00 mL·m^−2^. During spraying, the nozzle was positioned approximately 20.00 cm above the substrate surface, and each cycle lasted around 2 s. The coating was applied in three consecutive cycles. After spraying, the coated substrate was dried at 80 °C for 3 h to complete the film formation. A schematic diagram of the preparation process is shown in Figure 1.

To ensure material compatibility, the PFDT hydrophobic modifier was pretreated via solvent dilution and ultrasonic dispersion to achieve nanoscale uniformity prior to mixing. Meanwhile, EP was preheated to reduce viscosity and facilitate blending. For a homogeneous nanoparticle distribution within the EP matrix, hydrangea-like SiO_2_ nanoparticles were initially ultrasonically dispersed in the diluted PFDT modifier. Subsequently, preheated EP was gradually added under high-speed stirring (1000–1500 rpm), followed by continuous stirring for 30 min. A final ultrasonic treatment was conducted to eliminate agglomerates and ensure uniform dispersion throughout the mixture.

### 2.5. Characterization and Morphology

The elemental composition of the hydrangea-like mesoporous SiO_2_ and h-SiO_2_@PFDT superhydrophobic nanoparticles was analyzed using X-ray photoelectron spectroscopy (XPS, ESCALAB 250Xi, Thermo Fisher Scientific, Waltham, MA, USA). The characteristic functional groups of the nanoparticles were identified by Fourier transform infrared spectroscopy (FTIR, Nicolet 5700, Thermo Nicolet, Madison, WL, USA). The pore size distribution of the hydrangea-like mesoporous SiO_2_ nanoparticles was determined using a surface area and porosity analyzer (ASAP 2460, Micromeritics, Norcross, GA, USA). The morphology and internal structure of the nanoparticles were examined by transmission electron microscopy (TEM, Talos F200X, Thermo Scientific, Waltham, MA, USA). The surface morphology of both the nanoparticles and the coatings was characterized using field-emission scanning electron microscopy (FESEM, SU8020, Hitachi, Chiyoda-ku, Tokyo, Japan). The surface roughness and topography of the coatings were measured using a 3D optical profilometer (ContourGT-X, Bruker, Karlsruhe, Baden-Württemberg, Germany). The CA and SA of the coatings were measured using a contact angle goniometer (DSA 100, KRÜSS, Hamburg, Germany) to evaluate surface wettability.

### 2.6. Stability and Durability of Coatings

The mechanical stability of the coating was evaluated through a sandpaper abrasion test. A 500 g weight was affixed to a piece of 360-grit sandpaper (30 mm × 60 mm), which was dragged horizontally across the coated surface over a distance of 10 cm at a speed of approximately 2 cm·s^−1^, starting from one end of the coating. The chemical stability was assessed using acid and alkali immersion tests. Solutions with pH values ranging from 2 to 14 were prepared by diluting NaOH and HCl, and the coatings were immersed in these solutions at room temperature for 24 h. The changes in CA and contact angle (CA) were measured to evaluate any deterioration in surface wettability. For the artificial weathering test, the coatings were subjected to aging conditions for 30 days. During this period, the temperature and relative humidity were maintained at 40 ± 2 °C and 85 ± 5% RH, respectively. A UV lamp (365 nm wavelength, 50 W/m^2^ intensity) was employed for continuous illumination for 12 h per day to simulate solar radiation. Surface wettability was monitored after 30 days of artificial weathering and after immersion in a saline solution for 14 days.

### 2.7. Corrosion Resistance of Coatings

The anti-corrosion performance of the coating was evaluated using a VSP-300 (Yisi Qi (Beijing) Technology Development Co., Ltd., Beijing, China.) electrochemical workstation. A three-electrode electrochemical cell was configured, in which a 3.5 wt% NaCl solution was employed as the electrolyte and the superhydrophobic coating was used as the working electrode. Electrochemical impedance spectroscopy (EIS) was performed over a frequency range of 7 MHz to 10 mHz. The open-circuit corrosion potential (*E_corr_*) and corrosion current density (*I_corr_*) were determined by Tafel extrapolation. Based on *I_corr_*, the corrosion rate (*CR*) and inhibition efficiency (*η*) were calculated, where *CR* was determined using Equation (1) and *η* was obtained using Equation (2) [20]:

(1) $$CR=\frac{kM_{m}I_{\mathrm{corr}}}{\rho_{m}}$$

(2) $$\eta =\frac{I_{0}-I_{\mathrm{corr}}}{I_{0}}\;\times \;100\%$$

where *CR* represents the corrosion rate (mm·a^−1^), *k* is a constant equal to 3268.5, *M* is the molar mass of the metal (g·mol^−1^), and *ρ* is the density of the metal (g·cm^−3^). *I*_0_ denotes the corrosion current density of the bare aluminum alloy, while *I_corr_* corresponds to the corrosion current density of the superhydrophobic coating.

### 2.8. The Drag-Reduction Performance of Coatings

To ensure uniform spraying, a stable linear speed was applied to the substrate, and the distance between the spray gun and the outer surface of the pipeline was maintained at 0.5 m. The pipeline was rotated continuously at a constant speed to achieve an even coating deposition. Furthermore, a drag-reduction test was carried out on the solid outer pipe. The spraying of inner pipes can refer to the following methods. For the drag-reduction test of hollow metal pipe, the inner pipe wall with a large diameter (>150 mm) passes through a rotary air-spray gun (rod length = 1.5× pipe length); the speed is 30–60 rpm, the spray distance is 150–200 mm, the air pressure is 0.4–0.6 MPa, and the walking speed is 0.3 m/min. Medium-diameter (50–150 mm) pipes are prepared by using ultra-fine atomizing nozzles (atomized particle size ≤ 30 μm), sectional construction (each section ≤ 1 m), and sealing rotation (20 rpm) at both ends. The inner wall of the small-diameter (<50 mm) pipe can be using a supersonic cold-spraying robot via the aerosol spraying method. (carrier gas: He, pressure 1.5 MPa). The drag-reduction performance of the superhydrophobic coatings was evaluated using a custom-built drag-reduction testing apparatus, which primarily consisted of a sleeve-type testing channel and a data acquisition system. The pipeline was positioned horizontally, and upon opening the inlet valve, the fluid was allowed to flow along the inner surface of the pipe. Pressure-measuring ports were installed at both ends of the pipeline, and the pressure difference during fluid flow was recorded in real time by the data acquisition system. According to the Bernoulli equation, the flow resistance in a horizontal pipeline with a uniform diameter could be characterized by the pressure difference at a given flow rate. Therefore, the drag-reduction performance of the superhydrophobic coating was assessed by monitoring the pressure difference (Δ*p*) across both ends of the horizontal constant-diameter pipeline under identical flow conditions. To quantitatively evaluate the drag-reduction effect, a pressure drop ratio was introduced to calculate the drag-reduction rate; its mathematical expression is provided in Equation (3).

(3) $$DR=\frac{{\Delta p}_{\mathrm{bare}}-{\Delta p}_{\mathrm{coating}}}{{\Delta p}_{\mathrm{bare}}}\;\times \;100\%$$

where Δ*p_bare_* the differential pressure of flow in the bare inner tube and Δ*p_coating_* is the differential pressure of flow in the sprayed inner tube. The measurement was conducted three times to minimize the error.

## 3. Results and Discussion

### 3.1. h-SiO_2_ Nanoparticles and h-SiO_2_@PFDT—Characterization of Nanocomposite Materials

**Figure 2:**
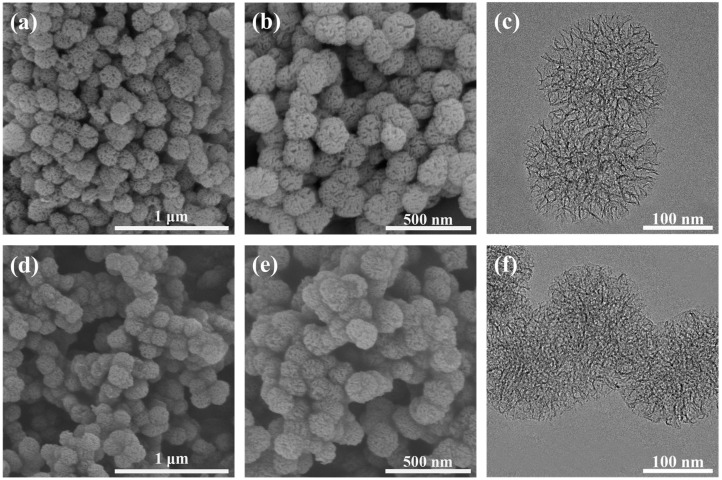
The SEM images of (**a**,**b**) h-SiO_2_ nanoparticles and (**d**,**e**) h-SiO_2_@PFDT nanocomposites under (**a**,**d**) low magnification and (**b**,**e**) high magnification, and TEM images of (**c**) h-SiO_2_ nanoparticles and (**f**) h-SiO_2_@PFDT nanocomposites.

The surface morphology and chemical composition of the synthesized h-SiO_2_ and h-SiO_2_@PFDT nanocomposites were investigated. As presented in Figure 2, the morphology of h-SiO_2_ and h-SiO_2_@PFDT nanomaterials was characterized by SEM and TEM. The SEM images of h-SiO_2_ nanoparticles (Figure 2a,b) revealed a hydrangea-like morphology with high monodispersity. The average particle size was approximately 200 nm, with a narrow size distribution, which ensured uniform dispersion within the EP matrix. Supporting these observations, TEM analysis confirmed the complex three-dimensional hydrangea architecture, as shown in Figure 2c.

The microporous characteristics of the h-SiO_2_ nanoparticles were further analyzed using nitrogen adsorption–desorption measurements (Figure S1). The h-SiO_2_ nanoparticles exhibited typical type IV isotherms with H1-type hysteresis loops. According to Table S1, the specific surface area was measured as 616.65 m^2^·g^−1^, with a dominant pore size of 10.37 nm. The high specific surface area and pore volume provided additional binding sites for PFDT, facilitating the grafting of hydrophobic groups and enhancing the superhydrophobic properties of the material. This unique porous structure also contributed to increased surface roughness, which is essential for achieving superhydrophobicity. The SEM images of h-SiO_2_@PFDT nanocomposites (Figure 2d,e) indicated that the distinctive hydrangea-like morphology of the original h-SiO_2_ nanoparticles was retained after PFDT surface modification. A continuous thin film was observed on the particle surfaces, linking adjacent particles, which was attributed to the hydrolysis and condensation of PFDT molecules, confirming successful surface grafting. TEM analysis (Figure 2f) further substantiated the formation of a network-like structure, indicating effective surface functionalization. The hydrangea-like SiO_2_ nanostructures were characterized by solid cores and wrinkled, petal-like outgrowths. This unique morphology increased the specific surface area and enhanced the adhesion of low-surface-energy modifiers, thereby improving both superhydrophobicity and structural durability. In addition, the folded features on the surface of the hydrangea nanoparticles not only increased the roughness of the coating surface but also provided more active sites for modifier grafting, enabling the h-SiO_2_@PFDT-EP superhydrophobic coating to trap a substantial amount of air. The trapped air film on the coating surface hindered the penetration of corrosive ions, thereby enhancing the anti-corrosion performance. Simultaneously, the air film promoted slip behavior at the liquid–solid interface, improving drag-reduction efficiency. These features were especially beneficial for marine applications, where reduced fluid friction and enhanced surface stability contributed to energy savings and a prolonged service life.

**Figure 3:**
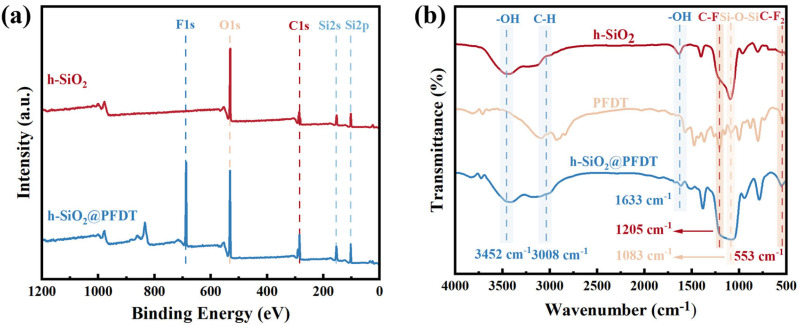
(**a**) XPS survey spectra of h-SiO_2_ nanoparticles and h-SiO_2_@PFDT nanocomposites. (**b**) FTIR spectra of h-SiO_2_ nanoparticles, PFDT, and h-SiO_2_@PFDT nanocomposites.

XPS analysis was conducted to determine the chemical composition of h-SiO_2_ and h-SiO_2_@PFDT nanoparticles. As shown in Figure 3a, the h-SiO_2_ nanoparticles were primarily composed of Si and O elements, with a small amount of surface carbon contamination. After surface modification with PFDT, the h-SiO_2_@PFDT particles were found to contain C, O, Si, and F elements, as illustrated in Figure S2. The presence of F indicated that PFDT (C_13_H_13_F_17_O_3_Si) had been successfully grafted onto the surface of h-SiO_2_ particles. Figure 3b presents the infrared spectra of h-SiO_2_, PFDT, and h-SiO_2_@PFDT. The characteristic absorption peak at 3008 cm^−1^ was attributed to the C–H stretching vibration. Broad absorption bands at 3452 cm^−1^ and 1633 cm^−1^ corresponded to the asymmetric stretching vibrations of –OH groups present on the surface of SiO_2_ particles. The absorption peak at 1083 cm^−1^ was assigned to the antisymmetric stretching vibration of Si–O–Si bonds. In comparison with unmodified h-SiO_2_, h-SiO_2_@PFDT exhibited two additional absorption peaks at 1205 cm^−1^ and 553 cm^−1^, corresponding to the stretching vibrations of C–F and C–F_2_ bonds in PFDT molecules, respectively. These findings confirmed that the surface of h-SiO_2_ nanoparticles was coated with PFDT, effectively lowering the surface free energy. Moreover, no –CH_2_ asymmetric stretching vibration peak of CTAB at 2851 cm^−1^ was detected in the infrared spectrum, indicating that CTAB had been completely removed during post-treatment and that impurity-related interference was eliminated.

### 3.2. Morphology and Wettability of Coatings

**Figure 4:**
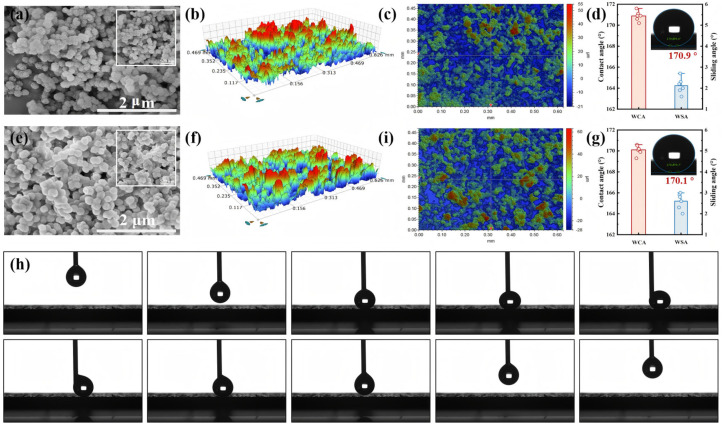
SEM images of (**a**) h-SiO_2_@PFDT and (**e**) h-SiO_2_@PFDT-EP coatings. Three-dimensional profiles of (**b**) h-SiO_2_@PFDT and (**f**) h-SiO_2_@PFDT-EP coatings. Vertical view of (**c**) h-SiO_2_@PFDT and (**i**) h-SiO_2_@PFDT-EP coatings. Contact angle and SA of (**d**) h-SiO_2_@PFDT and (**g**) h-SiO_2_@PFDT-EP, (**h**) Low-adhesion of a water droplet on superhydrophobic coatings.

The key to achieving a superhydrophobic surface lay in constructing an appropriate multiscale rough structure combined with low surface energy. The surface morphologies of the h-SiO_2_@PFDT and h-SiO_2_@PFDT-EP coatings were characterized by SEM, as shown in Figure 4. The SEM image of the h-SiO_2_@PFDT coating (Figure 4a) revealed that the spray-deposited surface exhibited abundant micro/nanostructures formed by the accumulation of nanoparticles. The corresponding 3D morphology (Figure 4b,c) displayed protrusions and depressions, with a randomly distributed topography composed of red peaks (protrusions) and blue valleys (pores). The h-SiO_2_@PFDT coating exhibited a high WCA of 170.9° and an SA of 2.0°, which were attributed to the synergistic effect between the hierarchical roughness and the low surface energy of the PFDT-modified nanoparticles. However, the weak physical adhesion among the particles rendered the coating susceptible to detachment under mechanical stress or prolonged use, which could potentially compromise its superhydrophobic performance.

To improve the mechanical durability and structural integrity, an appropriate amount of EP was incorporated into the h-SiO_2_@PFDT coating to form the h-SiO_2_@PFDT-EP coating. This modification enhanced the uniformity of surface roughness distribution and ensured strong interfacial bonding between the SiO_2_ nanoparticles and the EP matrix. SEM images of the h-SiO_2_@PFDT-EP coating (Figure 4e) at different magnifications revealed the presence of irregular surface textures and larger protrusions (>10 μm) originating from the micrometer-scale roughness of the epoxy bonding layer. These larger features were further composed of aggregated nanoscale h-SiO_2_@PFDT particles, thereby introducing additional roughness at the nanoscale. These results confirmed that the h-SiO_2_@PFDT-EP coating possessed hierarchical micro/nanostructures, satisfying the essential criteria for fabricating superhydrophobic surfaces.

Our 3D profilometry results (Figure 4f–i) demonstrated that, compared with the h-SiO_2_@PFDT coating, the h-SiO_2_@PFDT-EP coating exhibited a more homogeneous distribution of surface features and reduced roughness variation, further validating its enhanced wettability. The WCA of the h-SiO_2_@PFDT-EP coating remained nearly unchanged at 170.1°, while the SA decreased to 2.6°. Additionally, the adhesion properties of the h-SiO_2_@PFDT-EP coating were examined using contact angle goniometry by monitoring the behavior of water droplets during approach, contact, compression, and retraction. As shown in Figure 4h, even under compression, the droplets did not adhere to the surface, confirming the coating’s excellent low-adhesion performance.

The liquid repellency of the h-SiO_2_@PFDT-EP coating was investigated by applying various common liquids (e.g., pure water, ink, and tea) dropwise onto its surface. As shown in Figure S3, the wettability of the h-SiO_2_@PFDT-EP coating against different liquids was evaluated. All test droplets remained nearly spherical on the coating surface, indicating strong liquid repellency. The lowest WCA observed was 152.9° for oil droplets, while the WCA for all other tested liquids exceeded 157.1°. These results confirmed that the h-SiO_2_@PFDT-EP coating exhibited excellent repellency against a wide range of common liquids. The superhydrophobic performance of the h-SiO_2_@PFDT-EP coating was further evaluated by applying water droplets onto different substrates, including glass, paper, and plastic. As summarized in Table S2, the WCA on the plastic substrate was 155.5°, with a SA of 8.6°. For the other substrates, the WCA exceeded 161.9°, with SA values below 5°. These findings demonstrated that the h-SiO_2_@PFDT-EP coating maintained stable superhydrophobic properties across various substrate types.

### 3.3. Stability and Durability

**Figure 5:**
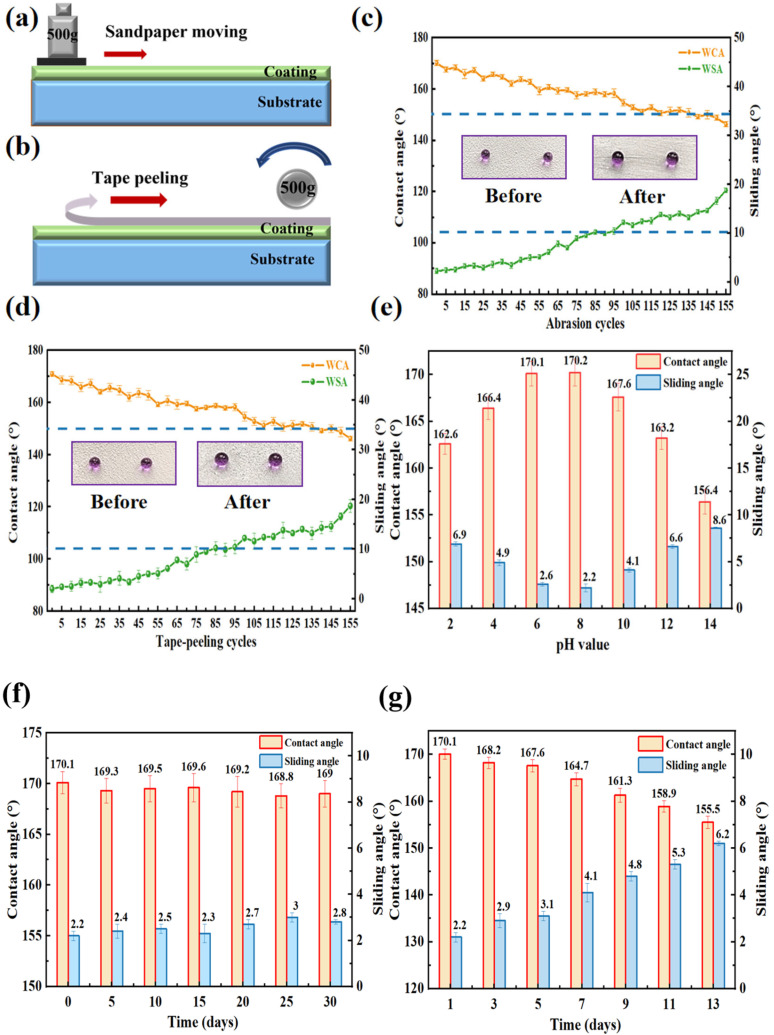
(**a**) Schematic diagram of the linear sandpaper abrasion test. (**b**) Schematic diagram of the repeated tape-peeling test. Changes in the wettability of the h-SiO_2_@PFDT-EP superhydrophobic coating under: (**c**) sandpaper abrasion, (**d**) tape peeling, (**e**) immersion in solutions with different pH values for 48 h, (**f**) artificial weathering for 30 days, and (**g**) salinization (NaCl solution) for 14 days.

The wear resistance of the h-SiO_2_@PFDT-EP superhydrophobic coating was evaluated using a linear sandpaper abrasion test (Figure 5a). As shown in Figure 5c, changes in the WCA and SA were recorded during the abrasion process. With an increasing number of abrasion cycles, the WCA gradually decreased while the SA increased. After 75 abrasion cycles, the WCA remained above 150.0° and the SA was still below 10.0°, indicating that the coating retained its superhydrophobic properties. Even after 105 cycles, although the SA exceeded 10.0°, the WCA remained above 150.0°, demonstrating strong wear resistance under linear mechanical stress.

The adhesion of the coating to the substrate was assessed through a repeated tape-peeling test (Figure 5b), and the corresponding wettability changes are depicted in Figure 5d. Initially, the h-SiO_2_@PFDT-EP coating exhibited a WCA of 170.1° and an SA of 2.2°. With an increasing number of tape peeling cycles, the WCA gradually decreased, and the SA slowly increased. After 80 cycles, the WCA dropped to 158.1°, while the SA increased to 9.5°, indicating that the coating still maintained excellent superhydrophobicity. Following 135 cycles, the WCA remained above 150.0° but the SA exceeded 10.0°, suggesting a partial loss of superhydrophobic behavior. These results confirmed that the h-SiO_2_@PFDT-EP coating exhibited strong adhesion to the substrate under mechanical stress.

A TEM image revealed a mesh-like structure that enhanced surface roughness and significantly improved particle adhesion, contributing to good mechanical durability. Additional experiments were conducted to investigate the mechanical stability and hardness of the h-SiO_2_@PFDT-EP coating, which exhibited excellent performance. As shown in Figure S4, the friction experienced by the coating increased gradually with higher applied loads. The maximum load sustained by the coating reached 75.36 N.

The chemical stability of the h-SiO_2_@PFDT-EP superhydrophobic coating was evaluated by immersing it in aqueous solutions with pH values of 2, 4, 6, 8, 10, 12, and 14 for 48 h. The coating’s surface wettability remained stable after extended acid and alkali exposure, as shown in Figure 5e. Specifically, the WCA of the coating remained above 150.0°, while the SA was maintained below 10.0°, indicating persistent superhydrophobicity. In strongly acidic conditions (pH = 2), the WCA reached 162.6°. Conversely, in highly alkaline environments (pH = 14), this value decreased to 156.4°; this still exceeds the threshold for superhydrophobic behavior.

The chemical stability of the h-SiO_2_@PFDT-EP superhydrophobic coating was evaluated by immersing it in aqueous solutions with pH values of 2, 4, 6, 8, 10, 12, and 14 for 48 h. As shown in Figure 5e, the coating’s surface wettability remained stable after prolonged exposure to both acidic and alkaline environments. Specifically, the WCA consistently remained above 150.0° and the SA was maintained below 10.0°, indicating sustained superhydrophobicity. Under strongly acidic conditions (pH = 2), the WCA reached 162.6°, while in highly alkaline conditions pH = 14), it decreased slightly to 156.4°; this value still exceeds the superhydrophobic threshold.

The weather resistance of the h-SiO_2_@PFDT-EP coating was examined by placing the coating in an indoor environment for 30 days. As shown in Figure 5f, the initial WCA and SA were 170.1° and 2.2°, respectively. After 30 days of natural aging, the WCA slightly decreased to 169.0°, and the SA increased marginally to 2.8°. These minor changes indicated that the coating maintained excellent superhydrophobic properties throughout the weathering period.

The salt resistance of the h-SiO_2_@PFDT-EP coating was evaluated by immersing the coating in a 3.5 wt% NaCl solution for 14 days. According to Figure 5g, the initial WCA and SA were 170.1° and 2.2°, respectively. After 14 days of saline immersion, the WCA remained at 155.5° and the SA was measured at 6.2°, confirming the coating’s durability and resistance to salinization.

The excellent environmental durability of the h-SiO_2_@PFDT-EP coating could be attributed to its hierarchical hydrangea-like nanostructure, which effectively trapped air and minimized direct contact between liquid and solid surfaces, thereby resisting chemical attack during weathering and salt exposure. In addition, the incorporation of EP enhanced the interparticle binding strength among h-SiO_2_@PFDT nanoparticles and improved the adhesion between the coating and the substrate. These combined effects contributed to the long-term structural integrity and stability of the superhydrophobic coating under various environmental stresses.

### 3.4. Corrosion Resistance

**Figure 6:**
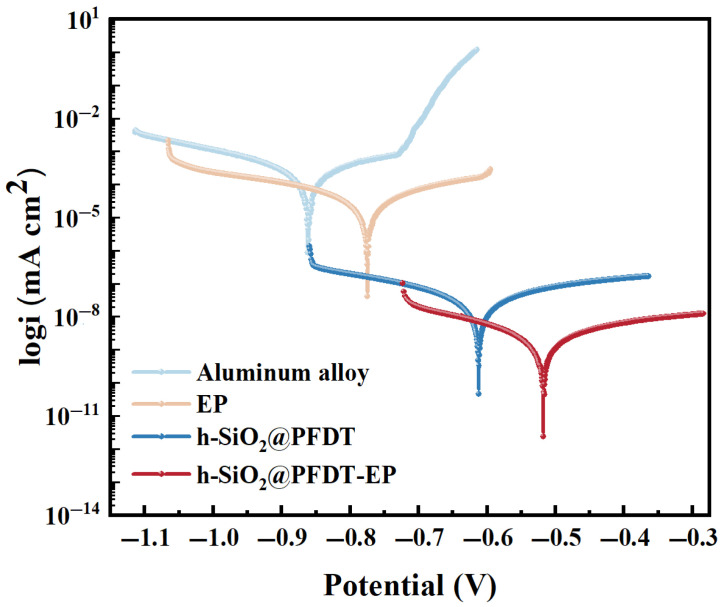
Polarization curves of bare aluminum alloy and different coatings.

**Figure 7:**
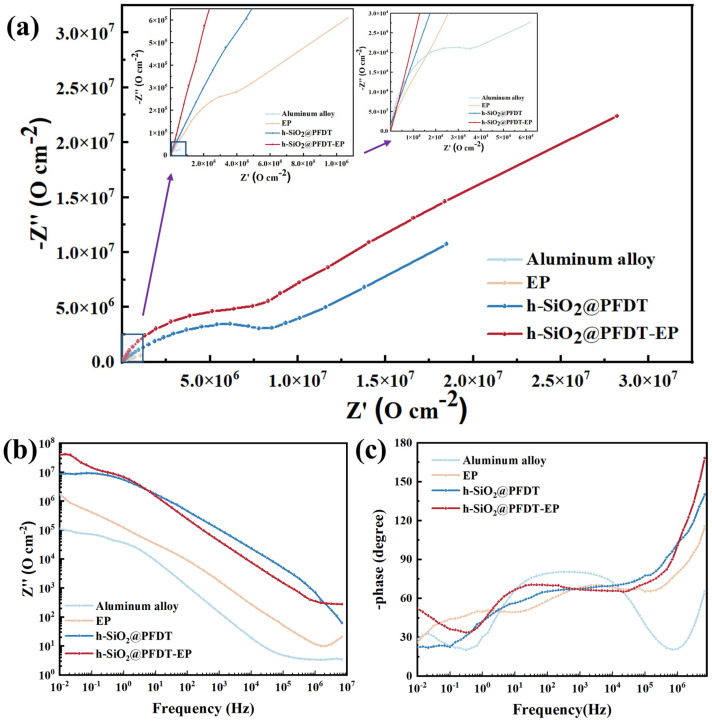
Nyquist curves of bare aluminum alloy and different coatings (**a**). (**b**) Low-frequency impedance modulus curve. (**c**) Phase-angle curve.

The corrosion resistance of the superhydrophobic coatings was evaluated using polarization curves and electrochemical impedance spectroscopy (EIS). Electrochemical corrosion tests were performed on 6061 aluminum alloy, EP, h-SiO_2_@PFDT, and h-SiO_2_@PFDT-EP coatings in a 3.5 wt% NaCl aqueous solution to assess their anti-corrosion performance. The polarization curves and impedance spectra for the bare alloy and the coated samples were experimentally measured, as shown in Figure 6 and Figure 7. Tafel extrapolation was employed to determine the corrosion potential (*Ecorr*) and corrosion current density (*Icorr*), with the corresponding values summarized in Table 2.

As shown in Figure 6 and Table 2, among the bare aluminum alloy and the three coating variants tested, the h-SiO_2_@PFDT-EP superhydrophobic coating exhibited the highest corrosion potential (*Ecorr* = −0.518 V) and the lowest corrosion current density (*Icorr* = 8.692 × 10^−10^ A·cm^−2^), indicating superior corrosion resistance. Compared to the uncoated 6061 aluminum alloy, the EP coating—commonly used as a protective barrier—demonstrated improved performance, with *Ecorr* positively shifted to −0.775 V and *Icorr* reduced by one order of magnitude, suggesting effective inhibition of electrochemical corrosion. The h-SiO_2_@PFDT coating, benefiting from its excellent superhydrophobicity, formed a stable air layer at the solid–liquid interface upon immersion, effectively preventing the penetration of water and corrosive ions. As a result, its *Ecorr* increased to −0.612 V and *Icorr* decreased to 1.169 × 10^−8^ A·cm^−2^. With the incorporation of the EP binder, the h-SiO_2_@PFDT-EP composite coating exhibited further enhancement in corrosion resistance, as *Ecorr* increased to −0.518 V and *Icorr* was reduced by four orders of magnitude compared to bare aluminum.

In addition, the calculated corrosion rate (CR) and inhibition efficiency (η) based on *Icorr* supported these findings. The h-SiO_2_@PFDT-EP coating showed the lowest corrosion rate, with a CR of only 2.79 × 10^−5^ mm·a^−1^, representing a reduction of 2–3 orders of magnitude compared to the EP and h-SiO_2_@PFDT coatings. The inhibition efficiency η reached as high as 99.92%. These results suggested a synergistic barrier and sealing effect between the hydrophobic nanostructures and the epoxy matrix that significantly enhanced the corrosion resistance of the h-SiO_2_@PFDT-EP coating. This conclusion was strongly supported by the polarization curve results.

Figure 7 presents the Nyquist and Bode plots for bare 6061 aluminum alloy and the three protective coatings. In general, a larger capacitive arc radius in the Nyquist plot and a higher impedance modulus (|Z|) at 0.01 Hz in the Bode plot indicate superior corrosion resistance. As shown in Figure 7a, the h-SiO_2_@PFDT-EP coating exhibited the largest capacitive arc among all samples, reflecting the highest charge transfer resistance and the most effective anti-corrosion performance. The order of arc radius followed the trend h-SiO_2_@PFDT-EP > h-SiO_2_@PFDT > EP > bare 6061 aluminum alloy.

The low-frequency impedance modulus |Z|_0.01_Hz is widely accepted as a direct indicator of coating corrosion resistance. As depicted in Figure 7b, the |Z| values of all coatings significantly exceeded that of the bare aluminum alloy at the same frequency. In particular, the |Z|_0.01_Hz value of the h-SiO_2_@PFDT-EP coating was the highest, surpassing that of the uncoated alloy by approximately two orders of magnitude, which was consistent with the Nyquist plot results.

Additionally, the phase angle Bode plot (Figure 7c) revealed that the bare aluminum alloy exhibited only a single time constant, indicating that its corrosion process was primarily governed by charge transfer at the metal/electrolyte interface. In contrast, the coated samples displayed two distinct time constants, indicating the presence of both barrier protection and interfacial processes. This behavior arose from the fact that the superhydrophobic coatings acted as effective dielectric barriers, impeding ion transport and thereby modifying the electrochemical kinetics. These results confirmed that the h-SiO_2_@PFDT-EP coating provided superior electrochemical stability and long-term protection in corrosive environments.

Moreover, the coating’s durability was evaluated through a 14-day immersion test, and the surface morphology after immersion is shown in Figure S5. No obvious pitting corrosion was observed, although the coating lost its superhydrophobicity. This result indicated that the coating possessed good long-term resistance to corrosive media on metal surfaces. The substantial decrease in *Icorr* by 3–4 orders of magnitude was likely due not only to the superhydrophobic effect but also to the synergistic contribution of the epoxy matrix.

### 3.5. Drag-Reduction Performance

**Figure 8:**
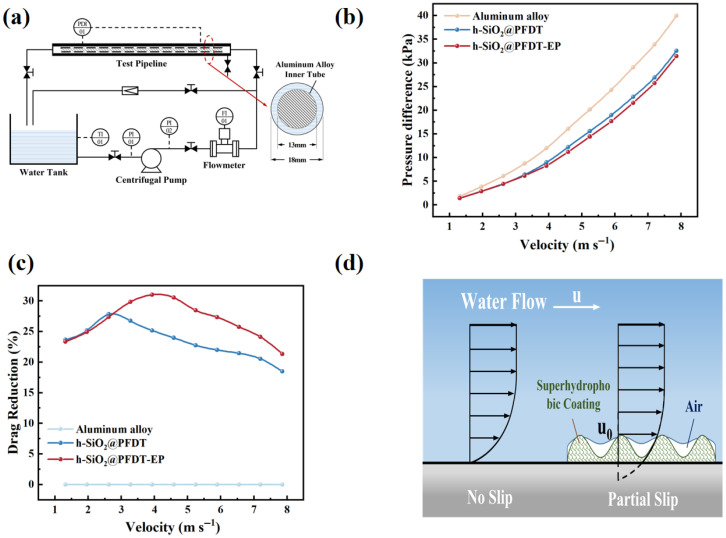
(**a**) Schematic diagram of the fluid-flow experimental setup. (**b**) Pressure drop variation of the superhydrophobic coatings at different flow velocities. (**c**) Drag-reduction rate of the superhydrophobic coatings as a function of flow velocity. (**d**) Schematic illustration of the drag-reduction mechanism of the h-SiO_2_@PFDT-EP superhydrophobic coating.

The drag-reduction performance of the h-SiO_2_@PFDT and h-SiO_2_@PFDT-EP superhydrophobic coatings was evaluated using a custom-built fluid dynamics testing system. The experiments were conducted by measuring the pressure difference (Δ*p*) at various flow rates to calculate the drag-reduction performance of the coatings. The Reynolds number during testing ranged from 2.8 × 10^3^ to 3.4 × 10^4^, corresponding to a turbulent flow regime. The relationship between the pressure difference and flow velocity is illustrated in Figure 8b, while the drag-reduction rate as a function of flow velocity is shown in Figure 8c. A schematic diagram of the experimental setup is provided in Figure 8d. The system primarily consisted of a water tank, centrifugal pump, test pipeline, and data acquisition instruments. The test section featured a detachable tube-in-tube structure, with a transparent PVE outer tube equipped with dual pressure-sensing ports at both ends. The inner tube was a sealed aluminum alloy cylinder supported by a 60° three-point frame at each end.

During testing, the inner tube was spray-coated with different superhydrophobic coatings and drag-reduction performance was evaluated by measuring pressure differentials. To ensure uniform coating coverage, the inner tube was divided into four 25 cm sections. Each section was sprayed for 30 s while masking the remaining areas with cardboard. The spray nozzle was positioned 30 cm from the surface, and the tube was rotated at 12 revolutions per minute. This segmented spraying method, combined with stable rotation, facilitated uniform coating deposition on the curved surface, which posed a greater challenge than flat surface application.

At lower flow velocities (1.31–2.62 m·s^−1^), the h-SiO_2_@PFDT coating demonstrated a higher drag-reduction rate than the h-SiO_2_@PFDT-EP coating, reaching a peak value of 27.83% at 2.62 m·s^−1^. However, as the velocity increased from 2.62 to 7.86 m·s^−1^, the h-SiO_2_@PFDT-EP coating exhibited superior drag-reduction performance, with a maximum rate of 31.01% at 3.93 m·s^−1^. As summarized in Table 3, both coatings effectively reduced fluid resistance, and the drag-reduction rate generally followed a trend of initially increasing and then decreasing with increasing flow velocity.

These observations could be explained by the intrinsic wettability and mechanical stability of the coatings. At low flow velocities, the h-SiO_2_@PFDT coating exhibited superior hydrophobicity, resulting in greater drag reduction through minimized liquid–solid contact. As flow velocity increased, the interfacial slip length also increased, further enhancing the drag-reduction effect. However, beyond a critical velocity, sustained shear stress and fluid impact progressively damaged the surface structures, leading to a degradation of hydrophobicity and a corresponding increase in drag.

In contrast, the addition of EP to the h-SiO_2_@PFDT-EP coating improved interparticle bonding and enhanced adhesion to the metal substrate, thereby increasing structural robustness under high-velocity conditions. As a result, the h-SiO_2_@PFDT-EP coating outperformed the h-SiO_2_@PFDT coating at elevated flow rates.

The drag-reduction mechanism is schematically illustrated in Figure 8a. For bare aluminum substrates, the no-slip condition dominated at the solid–liquid interface, resulting in high shear resistance. In contrast, the superhydrophobic coatings fabricated by cold spraying exhibited multiscale roughness—including protrusions, depressions, and pores—that effectively trapped air and formed a stable gas film at the interface. As shown in Figure S6, although the gas film on the superhydrophobic surface could not be directly observed during the experiment, its presence was indicated by the silver mirror phenomenon, which was caused by light reflection from the air layer formed by the micro/nanostructure. This phenomenon was absent on the bare 6061 aluminum substrate. The trapped gas layer significantly reduced the solid–liquid contact area and associated frictional drag. However, as the flow velocity increased, shear stress induced gradual fluid penetration into the surface roughness, compressing the gas film and increasing the contact area. Additionally, the surface roughness may have disrupted laminar flow, introducing form drag. These combined effects contributed to the decline in drag-reduction performance at higher flow velocities.

### 3.6. Antifouling Performance

**Figure 9:**
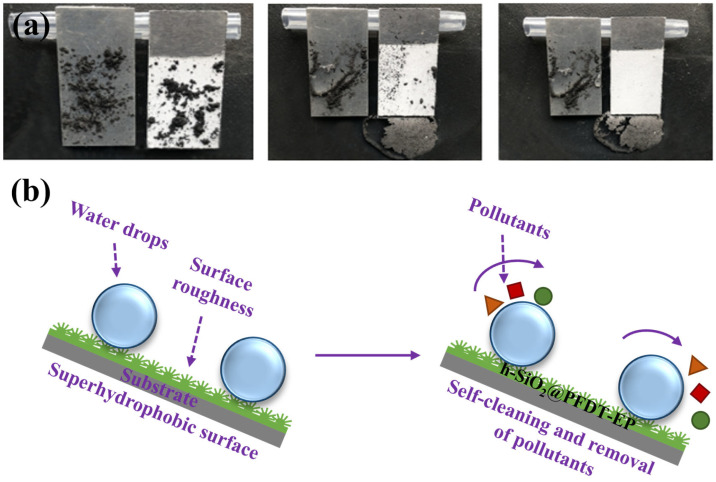
(**a**) Self-cleaning performance of the h-SiO_2_@PFDT-EP superhydrophobic coating demonstrated by the removal of graphite powder. (**b**) Schematic illustration of the self-cleaning mechanism of the h-SiO_2_@PFDT-EP superhydrophobic coating.

The antifouling performance of the h-SiO_2_@PFDT-EP superhydrophobic coating was evaluated by examining whether simulated pollutants (graphite powder) could be removed by rolling water droplets. As shown in Figure 9a, on the surface of bare 6061 aluminum alloy, water droplets coalesced into a single stream, adhered to the surface, and failed to remove the graphite powder. In contrast, on the h-SiO_2_@PFDT-EP superhydrophobic coating, water droplets rolled off along the inclined direction, effectively carrying away the graphite particles, demonstrating the coating’s excellent antifouling behavior. The self-cleaning mechanism is illustrated in Figure 9b. The surface of the h-SiO_2_@PFDT-EP coating featured a hierarchical micro/nanostructure constructed from hydrangea-like SiO_2_ particles and was further modified with low-surface-energy molecules. As a result, the solid–liquid contact area on the surface was minimized and water droplets adopted a near-spherical shape. This enabled the droplets to roll off easily, removing surface contaminants in the process. Therefore, the h-SiO_2_@PFDT-EP superhydrophobic coating exhibited outstanding self-cleaning and antifouling properties due to the synergistic effects of its hierarchical surface roughness and low surface energy.

## 4. Conclusions

In summary, a superhydrophobic coating based on h-SiO_2_@PFDT-EP was successfully fabricated and exhibited a high WCA of up to 170.1° and a low SA of 2.0°. The coating was composed of hydrangea-like SiO_2_ nanoparticles embedded in an EP matrix, with PFDT incorporated to reduce surface energy. The results demonstrated that the h-SiO_2_@PFDT-EP coating possessed excellent mechanical durability and chemical resistance, effectively withstanding abrasion (sandpaper wear), as well as exposure to acidic, alkaline, and saline environments. Moreover, the coating exhibited outstanding anti-corrosion and drag-reduction performance under underwater conditions. Its impedance modulus |Z| at 0.01 Hz remained approximately four orders of magnitude higher than that of bare 6061 aluminum alloy. Within a flow velocity range of 1.31 to 7.86 m·s^−1^, the drag-reduction rate reached a maximum of 31.01%. Based on experimental results, the coating was recommended for use under conditions such as an outdoor operating temperature of 40 ± 2 °C, a relative humidity below 85%, UV exposure for up to 30 days, and a maximum mechanical load of 75.36 N. This one-step spraying method provided a simple and efficient strategy for constructing superhydrophobic coatings using readily available and low-cost raw materials. The fabrication process was suitable for large-scale, large-area applications. Given its excellent performance, the h-SiO_2_@PFDT-EP superhydrophobic coating showed strong potential for practical use in a variety of fields, including drag reduction in marine vessels, pipeline transportation, and the anti-corrosion and antifouling protection of metal components in marine environments.

## Figures and Tables

**Table 1:** The compositions of 6061 aluminum alloy.

Elements	Si	Cu	Mg	Fe	Mn	Ti	Cr	Al
Mass fraction (wt.%)	0.36	0.02	1.74	0.30	0.07	0.02	0.20	97.29

**Table 2:** Self-corrosion voltage, self-corrosion current, corrosion rate, and inhibition rate of bare aluminum alloy and different coatings.

Samples	*E_corr_* (V)	*I_corr_* (A)	*CR* (mm·a^−1^)	*η* (%)
Aluminum alloy	−0.862	1.042 × 10^−6^	0.0334385	0
EP coating	−0.775	1.182 × 10^−7^	0.0037931	88.66
h-SiO_2_@PFDT coating	−0.612	1.169 × 10^−8^	0.0003751	98.38
h-SiO_2_@PFDT-EP coating	−0.518	8.692 × 10^−10^	0.0000279	99.92

**Table 3:** The drag-reduction rates of μ-SiO_2_@PFDT and h-SiO_2_@PFDT-EP coatings at different flow rates.

Velocity of Flow (m s^−1^)	h-SiO_2_@PFDT (%)	h-SiO_2_@PFDT-EP (%)
1.31	23.66	23.36
1.96	25.18	24.93
2.62	27.83	27.38
3.27	26.75	29.84
3.93	24.17	31.01
4.58	23.98	30.56
5.24	22.75	28.47
5.89	22.00	27.36
6.55	21.45	25.78
7.20	20.55	24.11
7.86	18.48	21.35

## Data Availability

The original contributions presented in this study are included in the article/Supplementary Materials. Further inquiries can be directed to the corresponding author.

## Supplementary Materials

The following supporting information can be downloaded at: https://www.mdpi.com/article/10.3390/ma18153443/s1, Table S1; BET specific surface area, BJH pore size distribution, and pore volume of mesoporous h-SiO_2_; Table S2: Water droplets in h-SiO2@PFDT-EP Wetting state of superhydrophobic material surface; Figure S1: Isothermal nitrogen adsorption desorption curve of mesoporous h-SiO_2_ particles (illustrated as pore size distribution); Figure S2: h-SiO2@PFDT EDS spectrum of particles; Figure S3: Various types of droplets are present h-SiO2@PFDT-EP Wetting state of superhydrophobic aluminum alloy surface; Figure S4: (a) Scratch test. (b) Photograph of the h-SiO2@PFDT-EP coating after the scratch test; Figure S5: The optical photo of the h-SiO2@PFDT-EP coating after being immersed in 3.5 wt% NaCl solution (a) 7days, (b) 14 days; Figure S6: Silver mirror phenomenon.
