# Supplementary material for: Durable Superhydrophobic Composite Coating Based on Hydrangea-like SiO2 Nanoparticles with Excellent Performance in Anticorrosion, Drag Reduction, and Antifouling
Source: Materials (Basel). 2025 Jul 23;18(15):3443. doi: 10.3390/ma18153443 (PMC12347627; doi:10.3390/ma18153443)
Supplement: Supplementary file 1 [file materials-18-03443-s001.zip › materials-3727094-supplementary.pdf]

Support Information

Durable superhydrophobic composite coating based on hydrangea-like SiO_2 nanoparticles with excellent performance in anticorrosion, drag reduction and antifouling

Yuhao Xue [1], Yamei Zhao [1,3,*], Xiaoqi Gu [1], Mengdan Huo[1], Kunde Yang [2], Mingyu Liu [1], Sixian Fan[1], Maoyong Zhi[3]

1 Department of Chemical Engineering, School of Environmental and Chemical Engineering, Xi'an Polytechnic University, Xi'an 710048, PR China

2 Ocean Institute of Northwestern Polytechnical University, Northwestern Polytechnical University, Taicang 215400, PR China

3 Sichuan Key Technology Engineering Research Center for All-electric Navigable Aircraft, Civil Aviation Flight University of China, Guanghan 618307, China

**** Corresponding authors.***

E-mail address: zhaoyameihp@126.com (Yamei Zhao)

Table S1.BET specific surface area, BJH pore size distribution, and pore volume of mesoporous h-SiO_2 particles.

Sample	S_{BET}(m^2 g^{-1})	D_{BJH}(nm)	V_{total}(cm^3 g^{-1})
h-SiO_2	616.65	10.37	1.68

Table S2. Water droplets in h-SiO_2@PFDT-EP Wetting state of superhydrophobic material surface.

Base material	Water contact angle(°)	Water sliding angle(°)
Glass	170.0	2.5
Aluminum alloy	169.6	2.7
Tin foil	166.1	3.4
Paper	165.0	3.5
Leather	163.7	3.8
Carbon fiber	163.2	3.9
Cotton	161.9	4.1
plastic	155.5	8.6

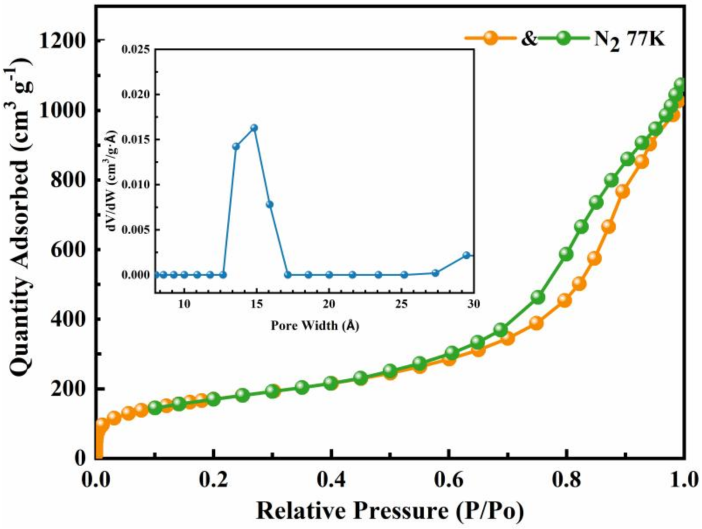

Fig. S1. Isothermal nitrogen adsorption desorption curve of mesoporous h-SiO_2 particles (illustrated as pore size distribution

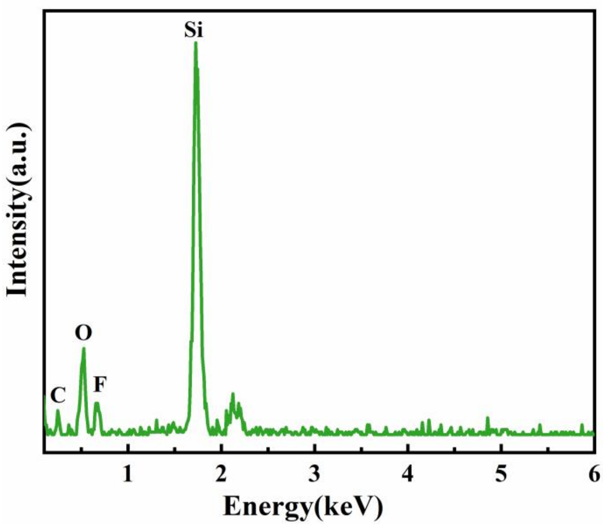

Fig. S2. h-SiO_2@PFDT EDS spectrum of particles.

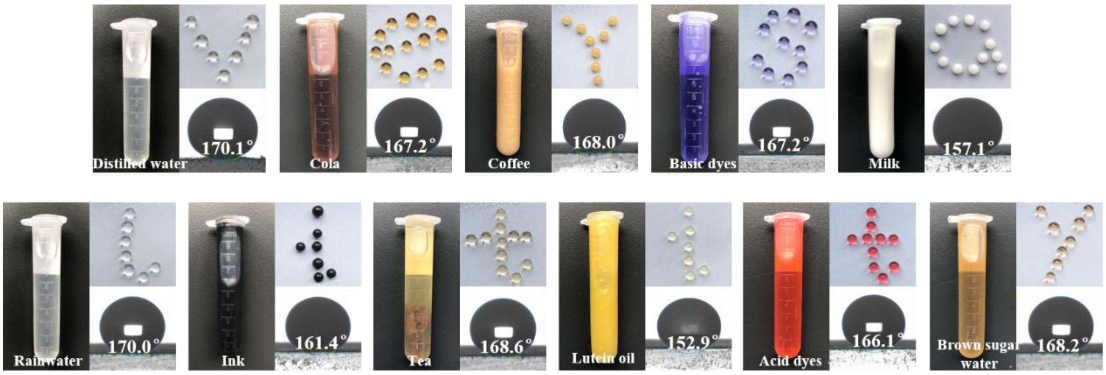

Fig. S3. Various types of droplets are present h-SiO_2@PFDT-EP Wetting state of superhydrophobic aluminum alloy surface.

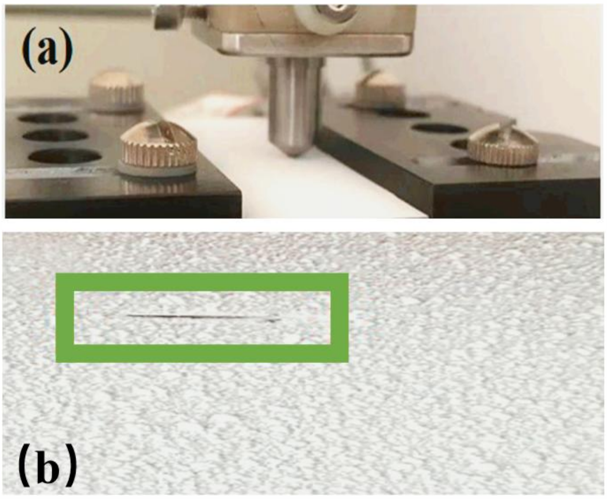

Fig. S4. (a) Scratch test. (b) Photograph of the h-SiO_2@PFDT-EP coating after the scratch test.

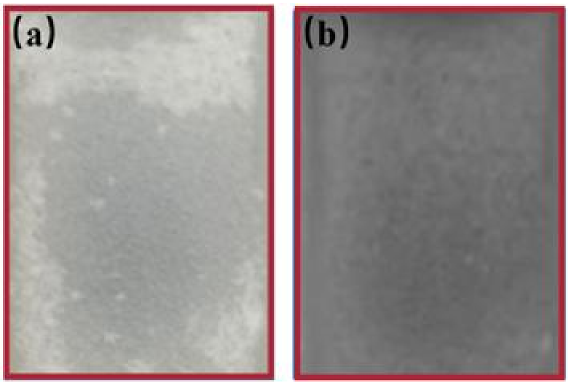

Fig. S5.The optical photo of the h-SiO_2@PFDT-EP coating after being immersed in 3.5 wt% NaCl solution.

（a）7 days,（b) 14 days

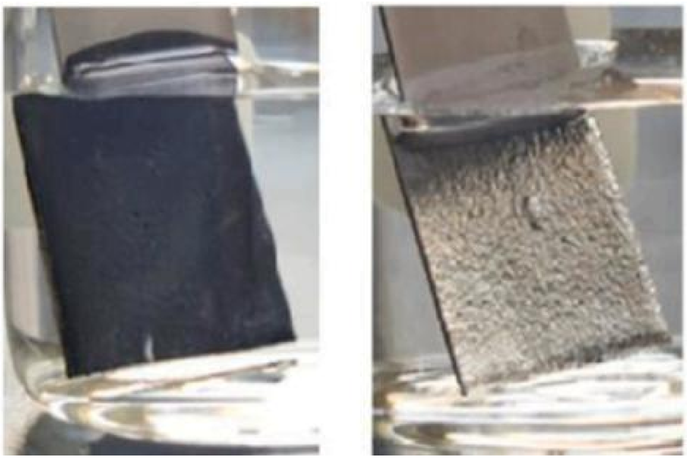

(1)Bare substrate (2) h-SiO_2@PFDT-EP Superhydrophobic coating

Fig.S6. Silver mirror phenomenon.
